# The Temporal Trends and Attributed Risk Burden of Kidney Cancer, Bladder Cancer, and Prostate Cancer in China From 1990 to 2021

**DOI:** 10.7759/cureus.87583

**Published:** 2025-07-09

**Authors:** Feng Gao, Li Yao, Jingfei Teng, Fuguang Zhao, Cong Ma, Xing Ai

**Affiliations:** 1 Department of Urology, The 7th Medical Center of Chinese People’s Liberation Army (PLA) General Hospital, Beijing, CHN; 2 Department of Urology, The 3rd Medical Center of Chinese People’s Liberation Army (PLA) General Hospital, Beijing, CHN

**Keywords:** bladder cancer, china, kidney cancer, prostate cancer, risk burden

## Abstract

Background: To analyze the temporal trends in incidence, prevalence, mortality, and disability-adjusted life-years (DALYs) of kidney, bladder and prostate cancers, and to quantify the attributed risk burden in China from 1990 to 2021.

Methods: The number and age-adjusted rate of incidence (age-standardized incidence rate (ASIR)), prevalence (age-standardized prevalence rate (ASPR)), mortality (age-standardized mortality rate (ASMR)) and DALYs (age-standardized DALY rate (ASDR)) for kidney, bladder and prostate cancers were presented in 2021 along with the change of age-standardized rates (ASRs) in China and globally by age and sex. The average annual percent change (AAPC) was shown and calculated by the Joinpoint regression program. The number of DALYs and ASDR for the three cancers attributed to smoking, high body mass index (BMI), high fasting plasma glucose (HFPG) and occupational exposure to trichloroethylene were presented from 1990 to 2021 by China.

Results: In 2021, bladder cancer had the highest cases of incidence (1.06×105, 95%UI: 0.83-1.37), mortality (0.45×105, 95%UI: 0.36-0.57) and DALYs (9.30×105, 95%UI: 7.35-11.85), while prostate cancer had the highest prevalence (6.28×105, 95%UI: 4.51-8.53). The largest growth in age-adjusted rate of incidence (ASIR) and prevalence (ASPR) was in prostate cancer, while the largest decrease in DALYs (ASDR) was in bladder cancer. The three cancers mainly occurred in men and elderly over 55 years old. The AAPC of ASIR and ASPR increased for the kidney (1.98, P<0.0001, 2.95, P<0.0001), bladder (0.3, P=0.038, 1.64, P<0.0001) and prostate cancers (2.4, P<0.0001, 3.97, P<0.0001); whilst the AAPC for ASMR and ASDR was insignificant. Finally, the risk burden of smoking, high BMI and HFPG consistently maintained a high level in China from 1990 to 2021.

Conclusion: This study describes the latest status and up-to-date burden of kidney, bladder and prostate cancers in China from 1990 to 2021, offering a comprehensive perspective on the policy making and intervention of these cancers.

## Introduction

Kidney, bladder and prostate cancers constitute the three common urologic cancers worldwide, and impose a significant burden on numerous people and societies globally [1]. For example, kidney and bladder cancers are identified as the 15th and 13th most common cancers in the world respectively, leading to more than 1,000,000 new cases and 490,000 deaths in 2020 [2,3]. Whilst, prostate cancer is ranked the second in prevalence and fifth in mortality in men all over the world [2,4].

The second commonality is that these urologic cancers are predominantly influenced by age, gender, tobacco exposure, and dietary patterns. Firstly, the incidence of these cancers is relatively high in developed countries, but the mortality is low. In contrast, developing countries have low incidence rates but higher mortality rates, especially in China [5-7]. Despite the treatment of cancer in China constantly improving and the five-year survival rate relatively increasing, these data are still behind those of developed countries [8].

The second commonality is that these urologic cancers are mainly influenced by the effects of age, gender, tobacco and dietary structure [9-11]. China has almost 20% of the world's population, which is facing aging of the population, obesity caused by the dietary structure, and a steady increase in the number of smokers [12-14]. However, the epidemiological trends in these urologic cancer burdens in China remain unknown. As China faces rapid aging of the population, rising obesity rates due to dietary westernization, and a smoking epidemic, all of which are established risk factors for urologic cancers, there remains a critical gap in comprehensive, long-term epidemiological data on kidney, bladder, and prostate cancers. Thus, in the present research, we aimed to estimate the up-to-date, long-term, and attributed burdens in the incidence, prevalence, mortality, and disability-adjusted life-years (DALYs) of these cancers in China.

## Materials and methods

Data source

The anonymized data in the present research were extracted from the Global Burden of Disease study 2021 (GBD 2021). Extraction parameters included selecting year from 1990 to 2021, causes with kidney cancer, bladder cancer and prostate cancer, smoking, high body mass index (BMI), high fasting plasma glucose (HFPG) and occupational exposure to trichloroethylene as the risk factor, and stratification by age (five-year bands), gender in China. GBD 2021 provided the estimates for the burden and attributed risk factors by age, sex for 371 diseases and injuries, and 87 risk factors in 204 countries and territories and 811 subnational locations from 1990 to 2021, which were harvested and further processed from literature, vital registration, surveys, censuses, administrative records, registries, individual studies, reports, and satellite imagery worldwide [15]. Our study included the incidence, prevalence, mortality (deaths) and DALYs both in numbers and age-standardized rates (ASRs) by age, sex and year and DALYs attributable to risk factors for kidney, bladder and prostate cancers in China. DALYs are an indicator for measuring the burden of disease, which is obtained by adding the years of life lost (YLL) due to premature death and the years lived with disability (YLD) due to disability.

Definition of diseases

GBD 2021 was mainly based on the International Classification of Diseases 10 (ICD-10). Kidney cancer was diagnosed by C64-C64.2, C64.4-C64.6, C64.8-C64.9, C65-C65.2, C65.9, D30.0-D30.1, and D41.0-D41.1, bladder cancer by C67-C67.9, D09.0, D30.3, D41.4-D41.8, D49.4, Z12.6-Z12.79, Z80.52, Z85.51, and prostate cancer by C61-C61.9, 185-185.9, V10.46, V16.42, V76.44 [16].

Risk factors

In GBD 2021, estimating risk factors has seven interconnected steps: 1) Estimate the effects through relative risk (RR) of health outcomes from the risk exposure; 2) Collect these exposure data and assess its distribution with Bayesian methods; 3) Set theoretical minimum risk exposure levels (TMRELs) based on epidemiological findings; 4) Calculate population-attributable fractions (PAFs) for every risk-outcome pair to estimate potential health improvement if exposure equals to TMREL; 5) Compute the age-specific exposure values (SEVs); 6) Rectify the PAFs through the mediation factors; 7) Multiply the PAFs with DALYs to estimate attributable risk burden [17].

In the present study, smoking was a common risk factor for kidney, bladder, and prostate cancers. Smoking status was defined as: (1) current smokers: individuals who used smoked tobacco products (cigarettes, cigars, pipes) daily or occasionally at the time of assessment; (2) former smokers: individuals who had quit smoking for six or more months prior to assessment, based on GBD 2021’s standardized questionnaire metrics. BMI and occupational exposure to trichloroethylene were associated with kidney cancer risk, while HFPG was linked to bladder cancer. High BMI was defined as BMI ≥28 kg/m2 for adults aged 20 or more years, while the range 20-23 kg/m2 in GBD 2021 reflects the TMREL. HFPG was defined as fasting plasma glucose ≥5.6 mmol/L, while the TMREL threshold of 4.9-5.3 mmol/L was based on global epidemiological studies. Occupational exposure to trichloroethylene was defined as self-reported historical exposure in industries such as metal degreasing, electronics manufacturing, or chemical synthesis, with exposure duration of three or more months and frequency of two or more days/week, as per GBD 2021’s occupational hazard classification system [17].

Statistical analysis

The data performed in our study included number and rate in metric for the incidence, prevalence, mortality and DALYs of the three cancers. Furthermore, the rates were shown as per 100,000 persons and ASRs were computed consistent with the GBD World Population Standard [18]. Joinpoint regression analysis was performed using Joinpoint Regression Program Version 4.9.0.0 (Surveillance Research Program, National Cancer Institute). The temporal trends of age-standardized incidence rate (ASIR), age-standardized prevalence rate (ASPR), age-standardized mortality rate (ASMR) and age-standardized DALY rate (ASDR) were analyzed by sex and age from 1990 to 2021. The number and position of joinpoints were determined using the Monte Carlo permutation test with 1,000 iterations and a significance level of P < 0.05, allowing up to five potential joinpoints. For each segment, the annual percent change (APC) was calculated, and the average annual percent change (AAPC) was derived as the geometric weighted average of APCs, weighted by the number of years in each segment. The model was fitted with a maximum of five joinpoints to balance model complexity and statistical significance, as recommended by the program’s default settings for trend analysis [6].

## Results

The status of kidney, bladder, and prostate cancers in 2021

In 2021, the incidences of kidney, bladder, and prostate cancers were 0.66×10^5 ^(95%UI: 0.54-0.80), 1.06×10^5^ (95%UI: 0.83-1.37) and 0.89×10^5^ (95%UI: 0.64-1.21) in China, accounting for 17.0%, 19.6% and 6.7% worldwide. The prevalence was 3.44×10^5^ (95%UI: 2.81-4.19), 5.70×10^5^ (95%UI: 4.51-7.36) and 6.28×10^5^ (95%UI: 4.51-8.53), accounting for 17.5%, 18.8% and 6.0%. More importantly, the mortality was 0.25×10^5^ (95%UI: 0.20-0.30), 0.45×10^5^ (95%UI: 0.36-0.57) and 0.37×10^5^ (95%UI: 0.28-0.50) accounting for 15.5%, 20.3% and 8.6%, while DALYs were 6.63×10^5^ (95%UI: 5.44-7.99), 9.30×10^5^ (95%UI: 7.35-11.85) and 6.81×10^5^ (95%UI: 5.05-9.34) for 16.5%, 21.2% and 8.4%. In addition, the ASIR, ASPR, ASMR and ASDR for the urological cancers in China were lower than those globally. However, the trends of ASIR, ASPR, ASMR and ASDR from 1990 to 2021 for kidney (0.85, 1.47, 0.09, -0.05) and prostate cancers (1.07, 2.23, 0.08, 0.06) in China were greater than those globally, while the trends of ASIR and ASPR for bladder cancer (0.1, 0.65) were greater, but those of ASMR (-0.32) and ASDR (-0.35) were lower than those in the world (Tables 1-3).

**Table 1:** Incidence, prevalence, mortality, and DALYs of kidney cancer in China compared with globe and distributed by age and sex. DALYs: disability-adjusted life-years, ASRs: age-standardized rates

Kidney cancer												
	Incidence			Prevalence			Mortality			DALYs		
Location	No in 100,000 (95UI) in 2021	ASRs per 100,000 (95UI) in 2021	ASRs change from 1990 to 2021	No in 100,000 (95UI) in 2021	ASRs per 100,000 (95UI) in 2021	ASRs change from 1990 to 2021	No in 100,000 (95UI) in 2021	ASRs per 100,000 (95UI) in 2021	ASRs change from 1990 to 2021	No in 100,000 (95UI) in 2021	ASRs per 100,000 (95UI) in 2021	ASRs change from 1990 to 2021
Global	3.88 (3.65,4.07)	4.52 (4.26,4.75)	0.16 (0.11,0.22)	19.61 (18.62,20.52)	22.70 (21.54,23.76)	0.32 (0.25,0.38)	1.61 (1.5,1.69)	1.91 (1.78,2.01)	-0.04 (-0.09,0)	40.16 (38.07,42.47)	47.33 (44.76,50.07)	-0.11 (-0.16,-0.05)
China	0.66 (0.54,0.80)	3.32 (2.73,3.98)	0.85 (0.48,1.32)	3.44 (2.81,4.19)	17.75 (14.63,21.29)	1.47 (0.97,2.14）	0.25 (0.20,0.30)	1.25 (1.03,1.48)	0.09 (-0.12,0.37)	6.63 (5.44,7.99)	34.18 (28.29,40.77)	-0.05 (-0.24,0.21)
Sex	No in 100,000 (95UI) in 2021	ASRs per 100,000 (95UI) in 2021	ASRs change from 1990 to 2021	No in 100,000 (95UI) in 2021	ASRs per 100,000 (95UI) in 2021	ASRs change from 1990 to 2021	No in 100,000 (95UI) in 2021	ASRs per 100,000 (95UI) in 2021	ASRs change from 1990 to 2021	No in 100,000 (95UI) in 2021	ASRs per 100,000 (95UI) in 2021	ASRs change from 1990 to 2021
Male	0.47 (0.36,0.59)	4.80(3.78,6.01)	1.08 (0.58,1.74)	2.44 (1.88,3.11)	24.82 (31.28,19.41)	1.98 (1.23,2.94)	0.18 (0.14,0.22)	1.92 (1.53,2.39)	0.22 (-0.07,0.58)	4.86 (3.77,6.18)	50.81 (40.15,63.77)	0.13 (-0.14,0.48)
Female	0.19 (0.14,0.25)	1.95 (1.44,2.52)	0.43 (-0.04,1.02)	1.00 (0.74,1.31)	10.70 (7.95,13.86)	0.76 (0.17,1.50)	0.07 (0.05,0.09)	0.69 (0.51,0.88)	-0.15 (-0.41,0.20)	1.78 (1.32,2.30)	18.46 (13.59,23.84)	-0.33 (-.05,-0.55)
Age	No in 100,000 (95UI) in 2021	ASRs per 100,000 (95UI) in 2021	ASRs change from 1990 to 2021	No in 100,000 (95UI) in 2021	ASRs per 100,000 (95UI) in 2021	ASRs change from 1990 to 2021	No in 100,000 (95UI) in 2021	ASRs per 100,000 (95UI) in 2021	ASRs change from 1990 to 2021	No in 100,000 (95UI) in 2021	ASRs per 100,000 (95UI) in 2021	ASRs change from 1990 to 2021
<20	0.02 (0.01,0.02)	0.53 (0.40,0.66)	-0.32 (-0.53,-0.05)	0.14 (0.11,0.17)	4.10 (3.14,5.11)	-0.24 (-0.47,0.07)	0.003 (0.002,0.004)	0.10 (0.07,0.12)	-0.68 (-0.79,-0.57)	0.28 (0.21,0.34)	8.27 (6.24,10.29)	-0.69 (-0.79,-0.57)
20-54	0.2 (0.16,0.25)	2.88 (2.32,3.57)	2.37 (1.58,3.41)	1.42 (1.15,1.75)	20.04 (16.19,24.69)	3.05 (2.13,4.29)	0.04 (0.04,0,.06)	0.63 (0.50,0.78)	0.75 (0.31,1.33)	2.06 (1.65,2.56)	29.10 (23.29,36.12)	0.70(0.28,1.25)
>55	0.44 (0.36,0.52)	11.50 (9.41,13.82)	1.13 (0.68,1.71)	1.88 (1.52,2.28)	49.71 (40.24,60.17)	2.32 (1.58,3.26)	0.2 (0.16,0.24)	5.30 (4.34,6.34)	0.36 (0.08,0.73)	4.29 (3.50,5.18)	113.39 (92.43,136.61)	0.27 (-0.01,0.63)

**Table 2:** Incidence, prevalence, mortality, and DALYs of bladder cancer in China compared with globe and distributed by age and sex. DALYs: disability-adjusted life-years, ASRs: age-standardized rates

Bladder cancer												
	Incidence			Prevalence			Mortality			DALYs		
Location	No in 100,000 (95UI) in 2021	ASRs per 100,000 (95UI) in 2021	ASRs change from 1990 to 2021	No in 100,000 (95UI) in 2021	ASRs per 100,000 (95UI) in 2021	ASRs change from 1990 to 2021	No in 100,000 (95UI) in 2021	ASRs per 100,000 (95UI) in 2021	ASRs change from 1990 to 2021	No in 100,000 (95UI) in 2021	ASRs per 100,000 (95UI) in 2021	ASRs change from 1990 to 2021
Global	5.40 (4.95,5.83)	6.35 (5.80,6.85)	-0.08 (-0.14,0.01)	30.26 (28.23,32.24)	34.91 (32.54,37.19)	0.04 (-0.02,0.14)	2.22 (2.01,2.42)	2.68 (2.42,2.93)	-0.23 (-0.29,-0.15)	43.97 (40.64,48.14)	51.58 (47.56,56.42)	-0.27 (-0.34,-0.18)
China	1.06 (0.83,1.37)	5.14 (4.08,6.62)	0.10 (-0.18,0.61)	5.70 (4.51,7.36)	26.61 (21.12,34.18)	0.65 (0.22,1.43)	0.45 (0.36,0.57)	2.34 (1.89,2.94)	-0.32 (-0.48,-0.02)	9.30 (7.35,11.85)	45.31 (36.06,57.41)	-0.35 (-0.51,-0.06)
Sex	No in 100,000 (95UI) in 2021	ASRs per 100,000 (95UI) in 2021	ASRs change from 1990 to 2021	No in 100,000 (95UI) in 2021	ASRs per 100,000 (95UI) in 2021	ASRs change from 1990 to 2021	No in 100,000 (95UI) in 2021	ASRs per 100,000 (95UI) in 2021	ASRs change from 1990 to 2021	No in 100,000 (95UI) in 2021	ASRs per 100,000 (95UI) in 2021	ASRs change from 1990 to 2021
Male	0.85 (0.64,1.13)	9.05 (6.89,11.95)	0.18 (-0.20,0.93)	4.63 (3.49,6.17)	44.62 (33.89,58.92)	0.78 (0.21,1.83)	0.35 (0.27,0.46)	4.32 (3.31,5.57)	-0.26 (-0.48,0.21)	7.37 (5.50,9.83)	78.58 (59.12,103.70)	-0.29 (-0.52,0.16)
Female	0.20 (0.16,0.26)	1.89 (1.44,2.39)	-0.21 (-0.44,0.11)	1.07 (0.83,1.36)	9.78 (7.58,12.41)	0.25 (-0.12,0.82)	0.10 (0.07,0.12)	0.92 (0.70,1.15)	-0.50 (-0.64,-0.31)	1.93 (1.48,2.45)	17.82 (13.66,22.57)	-0.52 (-0.66,-0.32)
Age	No in 100,000 (95UI) in 2021	ASRs per 100,000 (95UI) in 2021	ASRs change from 1990 to 2021	No in 100,000 (95UI) in 2021	ASRs per 100,000 (95UI) in 2021	ASRs change from 1990 to 2021	No in 100,000 (95UI) in 2021	ASRs per 100,000 (95UI) in 2021	ASRs change from 1990 to 2021	No in 100,000 (95UI) in 2021	ASRs per 100,000 (95UI) in 2021	ASRs change from 1990 to 2021
<20	0 (0,0)	0.02 (0.02,0.03)	-0.33 (-0.49,0.02)	0.006 (0.004,0.007)	0.17 (0.13,0.21)	-0.23 (-0.42,0.16)	0 (0,0)	0 (0,0)	-0.71 (-0.78,-0.57)	0.008 (0.007,0.01)	0.25 (0.19,0.31)	-0.70 (-0.77,-0.55)
20-54	0.16 (0.12,0.20)	2.20 (1.71,2.86)	0.67 (0.19,1.66)	1.21 (0.96,1.56)	17.05 (13.50,22.03)	1.02 (0.45,2.17)	0.03 (0.02,0.04)	0.43 (0.33,0.56)	-0.18 (-0.42,0.30)	1.42 (1.10,1.86)	20.02 (15.48,26.19)	-0.18 (-0.42,0.30)
>55	0.90 (0.71,1.16)	23.79 (18.81,30.69)	0.22 (-0.09,0.79)	4.49 (3.55,5.77)	118.52 (93.81,152.31)	0.79 (0.31,1.60)	0.42 (0.34,0.53)	11.1 (8.96,14.04)	-0.19 (-0.38,0.17)	7.87 (6.25,10.04)	207.74 (164.82,264.92)	-0.28 (-0.47,0.04)

**Table 3:** Incidence, prevalence, mortality, and DALYs of prostate cancer in China compared with globe and distributed by age and sex. DALYs: disability-adjusted life-years, ASRs: age-standardized rates

Prostate cancer												
	Incidence			Prevalence			Mortality			DALYs		
Location	No in 100,000 (95UI) in 2021	ASRs per 100,000 (95UI) in 2021	ASRs change from 1990 to 2021	No in 100,000 (95UI) in 2021	ASRs per 100,000 (95UI) in 2021	ASRs change from 1990 to 2021	No in 100,000 (95UI) in 2021	ASRs per 100,000 (95UI) in 2021	ASRs change from 1990 to 2021	No in 100,000 (95UI) in 2021	ASRs per 100,000 (95UI) in 2021	ASRs change from 1990 to 2021
Global	13.24 (12.17,14.0)	15.37 (14.13,16.25)	0.12 (0.08,0.17)	103.88 (97.06,109.04)	119.41 (111.69,125.27)	0.27 (0.21,0.32)	4.32 (3.82,4.64)	5.26 (4.65,5.64)	-0.17 (-0.21,-0.12)	81.42 (71.77,88.09)	95.94 (84.62,103.72)	-0.16 (-0.20,-0.11)
China	0.89 (0.64,1.21)	4.22 (3.01,5.73)	1.07 (0.53,1.73)	6.28 (4.51,8.53)	28.51 (20.48,38.50)	2.23 (1.39,3.26)	0.37 (0.28,0.50)	1.99 (1.47,2.69)	0.08 (-0.18,0.44)	6.81 (5.05,9.34)	33.69 (24.92,45.73)	0.06 (-0.21,0.41)
Sex	No in 100,000 (95UI) in 2021	ASRs per 100,000 (95UI) in 2021	ASRs change from 1990 to 2021	No in 100,000 (95UI) in 2021	ASRs per 100,000 (95UI) in 2021	ASRs change from 1990 to 2021	No in 100,000 (95UI) in 2021	ASRs per 100,000 (95UI) in 2021	ASRs change from 1990 to 2021	No in 100,000 (95UI) in 2021	ASRs per 100,000 (95UI) in 2021	ASRs change from 1990 to 2021
Male	0.89 (0.64,1.21)	4.22(3.01,5.73)	1.07 (0.53,1.73)	6.28 (4.51,8.53)	28.51 (20.48,38.50)	2.23 (1.39,3.26)	0.37 (0.28,0.50)	1.99 (1.47,2.69)	0.08(-0.18,0.44)	6.81 (5.05,9.34)	33.69 (24.92,45.73)	0.06(-0.21,0.41)
Female	0(0,0)	0(0,0)	0(0,0)	0(0,0)	0(0,0)	0(0,0)	0(0,0)	0(0,0)	0(0,0)	0(0,0)	0(0,0)	0(0,0)
Age	No in 100,000 (95UI) in 2021	ASRs per 100,000 (95UI) in 2021	ASRs change from 1990 to 2021	No in 100,000 (95UI) in 2021	ASRs per 100,000 (95UI) in 2021	ASRs change from 1990 to 2021	No in 100,000 (95UI) in 2021	ASRs per 100,000 (95UI) in 2021	ASRs change from 1990 to 2021	No in 100,000 (95UI) in 2021	ASRs per 100,000 (95UI) in 2021	ASRs change from 1990 to 2021
<20	0 (0,0)	0 (0,0)	0 (0,0)	0 (0,0)	0 (0,0)	0 (0,0)	0 (0,0)	0 (0,0)	0 (0,0)	0 (0,0)	0 (0,0)	0 (0,0)
20-54	0.06 (0.04,0.08)	0.81 (0.56,1.16)	2.83 (1.47,5.31)	0.53 (0.37,0.75)	7.46 (5.2,10.52)	3.19 (1.78,5.69)	0.008 (0.006,0.01)	0.12 (0.08,0.17)	0.17 (-0.26,0.97)	0.40 (0.28,0.56)	5.68 (3.96,7.85)	0.19 (-0.26,1.04)
>55	0.83 (0.59,1.13)	21.86 (15.62,29.90)	1.51 (0.82,2.34)	5.75 (4.11,7.77)	151.66 (108.53,205)	2.52 (1.59,3.68)	0.37 (0.27,0.49)	9.63 (7.17,12.99)	0.37 (0.02.0.81)	6.41 (4.79,8.80)	169.11 (126.38,232.18)	0.24 (-0.1,0.65)

Prostate cancer can only be suffered by men, and men were also more prone to the other two kinds of cancers compared with women. The ratios (men to women) of ASIR, ASPR, ASMR and ASDR were 2.46, 2.32, 2.78 and 2.75 for kidney cancer, while they were 4.79, 4.56, 4.70 and 4.41 for bladder cancer. The three types of urological cancers mainly occurred in the elderly over 55 years old, and the proportion of incidence, prevalence, mortality and DALYs were 66.7%, 54.7%, 82.3% and 64.7% for kidney cancer, while 84.9%, 78.7%, 93.3% and 84.6% for bladder cancer and 93.3%, 91.6%, 98.8% and 94.1%. Detailed results are shown in Tables 1-3.

The temporal trends of kidney, bladder, and prostate cancers in China

**Figure 1:**
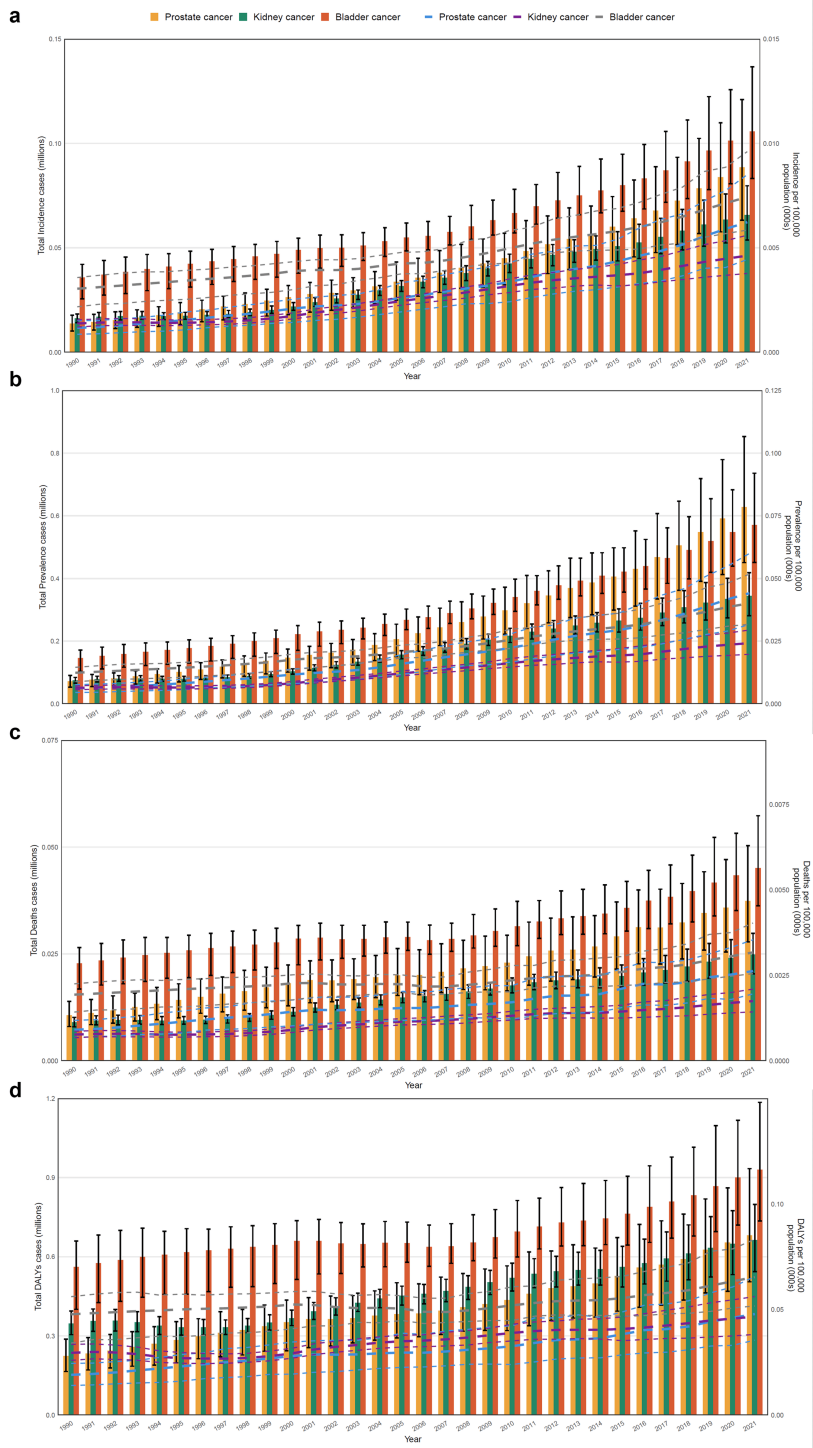
The temporal trends of incidence, prevalence, mortality and DALYs of kidney, bladder and prostate cancers. a. The annual distribution of the cases and the incidence rate for the three cancers. The cylinders represent the number of patients, and the dashed line represents the rate and its 95% UI. b. The annual distribution of the cases and the prevalence rate for the three cancers. c. The annual distribution of the cases and the mortality rate for the three cancers. c. The annual distribution of the cases and the DALYs rate for the three cancers. DALYs: disability-adjusted life-years

As shown in Figure 1, the cases of incidence and prevalence for three urological cancers increased from 1992 to 2021, while the extreme increases occurred after 2000. Furthermore, there was no significant change in the cases of mortality and DALYs from the three cancers before 2000. It showed a slight downward trend from 2000 to 2007, but there was a significant increase from 2007 to 2021.

To better understand the temporal trends of ASRs for three cancers, we used the APC model to divide segments and elucidate the overall trend by citing the AAPC values from 1990 to 2021 in China. The AAPC of ASIR and ASPR showed increased for the kidney (1.98, P<0.0001, 2.95, P<0.0001), bladder (0.3, P=0.038, 1.64, P<0.0001) and prostate cancers (2.4, P<0.0001, 3.97, P<0.0001) respectively. These data showed prostate cancer increased the most in ASIR and ASPR, while bladder cancer increased the least. Furthermore, despite the insignificant AAPC of ASMR and ASDR, these data showed that the AAPC of ASMR for kidney (2.15, P=0.87) and prostate cancers (1.59, P=0.78) showed an increase from 1990 to 2021, while that for bladder cancer decreased (-4.45, P=0.78). Moreover, the AAPC of ASDR for bladder (0.54, P=0.94) and prostate cancers (0.18, P=0.96) showed a slight increase, while that for kidney cancer decreased (-0.89, P=0.86). Detailed results are shown in Table 4.

**Table 4:** The APC trends and AAPC of ASIR, ASPR, ASMR, and ASDR for kidney, bladder and prostate cancer in China from 1990 to 2021. APC: annual percent change, AAPC: average annual percent change, ASIR: age-standardized incidence rate, ASPR: age-standardized prevalence rate, ASMR: age-standardized mortality rate, ASDR: age-standardized DALY rate, DALYs: disability-adjusted life-years

Cancer type	Measures	APC(95%CI)						AAPC(95%CI)	P value
Kidney cancer	ASIR	1990-1997	1997-2002	2002-2005	2005-2011	2011-2016	2016-2021	1.98(1.66,2.3)	<0.0001
		-0.31(-0.66,0.04)	4.70(3.97,5.42)	3.88(1.77,6.03)	2.67(2.15,3.20)	0.49(-0.47,1.47)	2.08(1.08,3.10)		
	ASPR	1990-1992	1992-1995	1995-1998	1998-2002	2002-2009	2009-2021	2.95(2.52,3.89)	<0.0001
		2.20(-1.10,5.61)	-1.07(-3.83,1.77)	2.36(-0.07,4.85)	7.07(5.88,8.28)	4.44(4.06,4.83)	2.05(1.83,2.27)		
	ASMR	1990-1996	1996-1999	1999-2011	2011-2014	2014-2017	2017-2021	2.15 (-20.32,30.96)	0.8666
		13.85(-3.99,35.02)	-22.06(-85.52,319.59)	0.98(-6.17,8.67)	23.09(-82.18,750.05)	-70.93(77.75,-0.78)	24.60(-11.0,74.45)		
	ASDR	1990-1992	1992-1996	1996-2001	2001-2005	2005-2019	2019-2021	-0.89 (-10.41,9.63)	0.8616
		-21.09(-68.71,98.99)	2.77(-20.11,32.19)	-1.06(-24.84,30.25)	0.89(-18.83,25.41)	-0.63(-3.45,2.27)	10.11 (-61.11,211.73)		
Bladder cancer	ASIR	1990-1995	1995-2000	2000-2007	2007-2011	2011-2016	2016-2021	0.3(0.02,0.58)	0.0375
		0.56(-0.15,1.28)	-0.15(-1.04,0.76)	-0.77(-1.12,-0.42)	1.33(0.20,2.49)	0.001(-0.77,0.77)	1.45(0.70,2.20)		
	ASPR	1990-1997	1997-2000	2000-2007	2007-2011	2011-2016	2016-2021	1.64(1.38,1.91)	<0.0001
		1.42(1.08,1.76)	2.12(-0.07,4.36)	1.02(0.73,1.31)	2.69(1.84,3.54)	0.98(0.40,1.57)	2.38(1.79,2.97)		
	ASMR	1990-1992	1992-2001	2001-2004	2004-2012	2012-2015	2015-2021	-4.45 (-30.94,32.21)	0.7836
		-34.04(-88.81,288.8)	-4.42(-16.94,1.0)	25.90(-88.25,1249.44)	-9.62(-22.14,4.91)	51.93(-85.99,1548.12)	-19.58 (-37.91,41.5)		
	ASDR	1990-1992	1992-1996	1996-2001	2001-2015	2015-2019	2019-2021	0.54 (-12.75,15.85)	0.9409
		27.6(-62.81,337.85)	-5.32(-52.66,89.35)	0.57(-18.39,23.95)	-0.75(-5.2,3.91)	3.37(-29.99,52.65)	-7.61 (-70.29,187.31)		
Prostate cancer	ASIR	1990-1992	1992-1995	1995-2000	2000-2003	2003-2017	2017-2021	2.4(1.96,2.84)	<0.0001
		2.71(-0.14,5.64)	4.53(1.61,7.54)	3.13(2.22,4.06)	0.65(-2.01,3.38)	1.97(1.83,2.13)	2.57(1.54,3.61)		
	ASPR	1990-2000	2000-2003	2003-2007	2007-2013	2013-2016	2016-2021	3.97(3.58,4.36)	<0.0001
		4.73(4.53,4.93)	2.52(0.05,5.04)	5.70(4.43,6.98)	3.33(2.76,3.90)	1.78(-0.78,4.40)	4.04(3.33,4.76)		
	ASMR	1990-1993	1993-1996	1996-2007	2007-2013	2013-2016	2016-2021	1.59(-9.07,13.51)	0.7798
		37.68(-7.47,104.74)	-27.08(-64.22,48.62)	5.29(-0.49,11.40)	-11.28(-23.76,3.23)	44.76(-34.37,219.27)	-9.13 (-26.63,12.54)		
	ASDR	1990-1994	1994-1999	1999-2003	2003-2007	2007-2019	2019-2021	0.18(-6.19,6.99)	0.9561
		0.81(-7.82,10.24)	-1.95(-20.24,20.54)	1.72(-16.7,24.23)	-1.04(-27.49,35.06)	0.002(-3.43,3.56)	4.95(-41.34,87.76)		

In order to better evaluate the mortality for the three types of cancer, we have cited the mortality-to-incidence rate (MIR). The temporal trend of MIR for the kidney (0.64 to 0.38), bladder (0.73 to 0.45) and prostate cancers showed a decrease from 1990 to 2021 in China. Prostate cancer had the highest MIR in 1990 and 2021 and decreased most (0.9 to 0.47), and bladder cancer had the second highest MIR and decreased from 0.73 to 0.45, while kidney cancer had the lowest and decreased from 0.64 to 0.38. Detailed results are shown in Table 5.

**Table 5:** The temporal trends of ASIR, ASMR and MIR. MIR were calculated by ASMR to ASIR. ASIR: age-standardized incidence rate, ASMR: age-standardized mortality rate, MIR: mortality-to-incidence rate

Year	Kidney cancer			Bladder cancer			Prostate cancer		
	ASIR	ASMR	MIR	ASIR	ASMR	MIR	ASIR	ASMR	MIR
1990	1.79	1.14	0.64	4.69	3.44	0.73	2.04	1.84	0.90
1991	1.83	1.15	0.63	4.73	3.44	0.73	2.08	1.85	0.89
1992	1.84	1.15	0.63	4.77	3.44	0.72	2.15	1.91	0.89
1993	1.83	1.14	0.62	4.80	3.43	0.72	2.25	1.98	0.88
1994	1.78	1.10	0.62	4.81	3.41	0.71	2.34	2.04	0.87
1995	1.78	1.09	0.61	4.84	3.40	0.70	2.45	2.12	0.86
1996	1.80	1.09	0.60	4.84	3.36	0.69	2.54	2.16	0.85
1997	1.82	1.08	0.60	4.81	3.30	0.69	2.61	2.17	0.83
1998	1.86	1.09	0.59	4.80	3.26	0.68	2.66	2.18	0.82
1999	1.93	1.12	0.58	4.80	3.22	0.67	2.76	2.23	0.81
2000	2.04	1.17	0.58	4.86	3.23	0.67	2.86	2.27	0.79
2001	2.18	1.24	0.57	4.81	3.17	0.66	2.92	2.29	0.78
2002	2.26	1.25	0.55	4.67	3.01	0.65	2.87	2.18	0.76
2003	2.32	1.26	0.54	4.63	2.95	0.64	2.88	2.14	0.74
2004	2.43	1.27	0.52	4.67	2.90	0.62	2.96	2.12	0.72
2005	2.52	1.28	0.51	4.69	2.81	0.60	3.06	2.09	0.68
2006	2.59	1.26	0.49	4.56	2.63	0.58	3.10	2.02	0.65
2007	2.65	1.25	0.47	4.56	2.57	0.56	3.18	2.02	0.63
2008	2.73	1.26	0.46	4.60	2.54	0.55	3.24	2.00	0.62
2009	2.80	1.26	0.45	4.65	2.51	0.54	3.25	1.94	0.60
2010	2.88	1.27	0.44	4.74	2.51	0.53	3.31	1.92	0.58
2011	2.95	1.28	0.43	4.80	2.50	0.52	3.44	1.99	0.58
2012	2.98	1.27	0.43	4.82	2.46	0.51	3.54	2.02	0.57
2013	2.98	1.23	0.41	4.80	2.39	0.50	3.54	1.94	0.55
2014	2.99	1.21	0.40	4.77	2.34	0.49	3.56	1.91	0.54
2015	3.00	1.22	0.41	4.79	2.36	0.49	3.68	2.03	0.55
2016	3.02	1.23	0.41	4.83	2.38	0.49	3.79	2.10	0.55
2017	3.08	1.21	0.39	4.85	2.33	0.48	3.81	1.98	0.52
2018	3.17	1.22	0.39	4.92	2.31	0.47	3.90	1.96	0.50
2019	3.26	1.24	0.38	5.02	2.33	0.46	4.06	2.01	0.49
2020	3.28	1.24	0.38	5.10	2.34	0.46	4.15	1.99	0.48
2021	3.32	1.25	0.38	5.14	2.34	0.45	4.22	1.99	0.47

The temporal trends of attributed risk burden of kidney, bladder, and prostate cancers in China from 1990 to 2021

**Figure 2:**
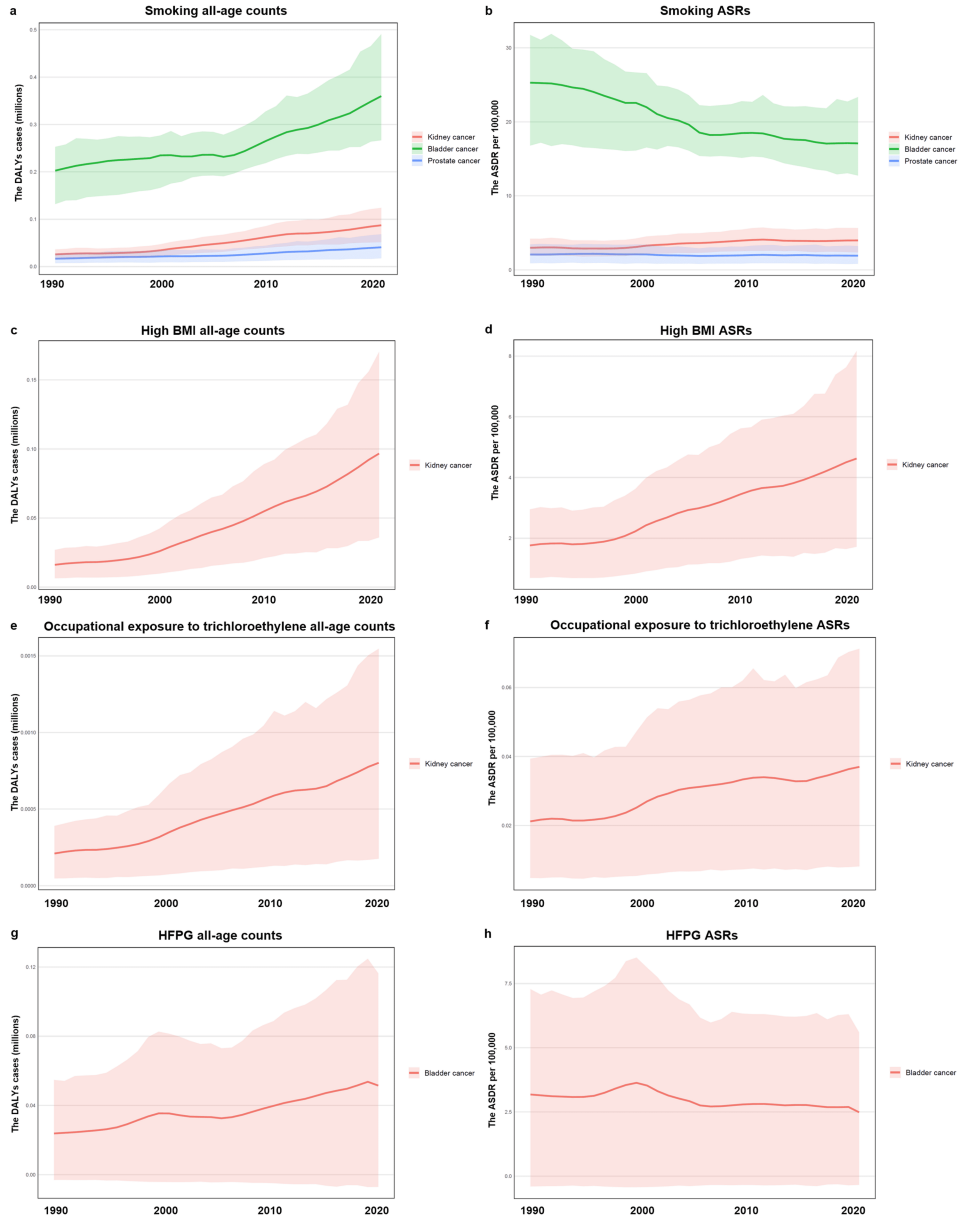
The cases and ASRs of different risk factors for kidney, bladder and prostate cancers. a. The cases of DALYs attributed to smoking for kidney, bladder and prostate cancers. b. The ASRs of DALYs attributed to smoking for kidney, bladder and prostate cancers. c. The cases of DALYs attributed to high BMI for kidney cancer. d. The ASRs of DALYs attributed to high BMI for kidney cancer. e. The cases of DALYs attributed to occupational exposure to trichloroethylene for kidney cancer. f. The ASRs of DALYs attributed to occupational exposure to trichloroethylene for kidney cancer. g. The cases of DALYs attributed to HFPG for bladder cancer. h. The ASRs of DALYs attributed to HFPG for bladder cancer. DALYs: disability-adjusted life-years, ASRs: age-standardized rates, HFPG: high fasting plasma glucose, BMI: body mass index

As shown in Figure 2, the DALYs attributed to smoking showed an increasing trend from 1990 to 2021 for kidney (25,803-87,270), bladder (202,646-359,985) and prostate cancers (16,742-40,781) in China. The ASRs attributed to smoking for bladder cancer decreased most (25.29%-17.09%), and prostate cancer decreased slightly (2.08%-1.92%), while kidney cancer increased slightly (2.99%-3.98%). The DALYs attributed to high BMI were 16,153 to 96,675 from 1990 to 2021, while the ASRs were 1.76% to 4.62% in China. The DALYs attributed to high BMI for kidney cancer were 16,153 to 96,675 from 1990 to 2021, while the ASRs were 1.76% to 4.62% in China. Furthermore, the DALYs attributed to occupational exposure to trichloroethylene were 210 to 801, while the ASRs were 0.02% to 0.04% in China from 1990 to 2021. Finally, the DALYs attributed to HFPG for bladder cancer were 25,031 to 24,515 from 1990 to 2021, while the ASRs were 3.18% to 2.49% in China.

## Discussion

Our current study comprehensively explored the temporal trends and attributed risk burden of incidence, prevalence, mortality and DALYs for kidney, bladder and prostate cancers from 1990 to 2021 in China based on the GBD 2021 platform. Despite the lower ASRs compared with the global averages, the ASIR, ASPR, ASMR, and ASDR for the three cancers (except for the ASMR and ASDR of bladder cancer) all showed upward trends and had higher average growth rates than the global levels from 1990 to 2021. Furthermore, there were significant differences in the burden of the three cancers among men and people aged 55 and above. Finally, although the ASRs attributed to smoking for bladder and prostate cancers decreased, the cases attributed to smoking and the cases and ASRs to other factors still showed an increased trend in China. Our present research will provide a theoretical basis for the formulation of policies and reinforcement of interventions for urological cancers in China.

As the most populous country, China accounts for 17.72% of the world's population [19]. Although the incidence, prevalence, mortality and DALYs of the three types of cancer in China are lower than the global average level, their growth trends are higher than the global level, which is supported by previous studies [6,20,21]. Furthermore, the current study indicated that the disease burden of the three cancers is mainly concentrated among people aged 55 and above. The ageing population of China is continuously increasing, leading to the cases of urological cancers continuing to rise, which will impose a severe burden on public health security. Notably, the MIR in China showed a decreased trend from 1990 to 2021, especially the bladder and prostate cancers which had decreased trends of ASMR and ASDR and the greatest decrease in MIR, respectively. These might be mainly attributed to the continuous development of immunotherapy and targeted therapy, for example tyrosine kinase inhibitors for kidney cancer [22], inhibitor of fibroblast growth factor receptor (FGFR) for bladder cancer [23] and targeted therapy for prostate-specific membrane antigen (PSMA) for prostate cancer [24]. Furthermore, the increase in the incidence can lead to an increase in prevalence, and the reason for this may be the continuous improvement of diagnostic techniques in China in recent years [1].

Smoking is a common risk factor for these urological cancers, especially bladder cancer [1,5,6,21]. Our current study indicated that the cases of DALYs for the three cancers attributed to smoking showed an increasing trend in China from 1990 to 2021, which was also supported by the previous study [5,6,20,21]. Based on recent studies from Chinese serial cross-sectional National Health Service Surveys, the prevalence of tobacco use in China is consistently high with a proportion of 25% from 2003 to 2013, while male smokers stayed at approximately 20 times than female smokers [25]. Moreover, the attributed risk burden of the high BMI for kidney cancer and the HFPG for bladder cancer were also increased in China. These might be attributed to the Chinese dietary structure continuously westernized in recent years [9,10]. Finally, the proportion of kidney cancer cases caused by occupational exposure to trichloroethylene is very small as shown in our current study. However, this proportion has been continuously increasing over time, which should draw the attention of Chinese policymakers.

In our present research, there are obvious gender discrepancies in the cases and ASRs of incidence, prevalence, mortality and DALYs for the three cancers in China. These might be partly attributed to the risk burden such as the huge gap in the proportion of smokers between genders as mentioned above. Biologically, androgen-driven prostate epithelial cell proliferation underlies prostate cancer’s exclusive male occurrence [26], while testosterone may promote bladder cancer progression via androgen receptor-mediated signaling [27]. Additionally, there are differences in the social division of labor between men and women. Men may face more dangerous working environments [28]. Finally, there are physiological differences between men and women, which may make men more prone to urinary system cancers. Therefore, the disparities between genders must be taken into account when formulating and implementing population-wide prevention strategies and tailoring individualized treatment plans for urological cancers.

The present research still has some limitations. Firstly, the data retracted from the GBD 2021 platform were computed based on a Bayesian meta-regression model named DisMod-MR 2.1 from various sources including literature, vital registration, surveys, censuses, administrative records, registries, individual studies, reports, and satellite imagery around world, which may lead to deviations from the epidemiological investigations conducted locally in China [1,15]. Moreover, the GBD 2021 platform only provides the attributed risk burdens including smoking, high BMI, HFPG, occupational exposure to trichloroethylene, and so on. These risk factors cannot fully assess the risk of cancer occurrence. Finally, being influenced by the ecological fallacy was unavoidable. This was due to the fact that the interpretations of the results were grounded in population-level data rather than that of individual-level. As a result, additional studies based on individual cases should be carried out to verify the findings of this research.

## Conclusions

Our present study performed a comprehensive analysis for temporal trends and attributed burdens for the kidney, bladder and prostate cancers in China from 1990 to 2021. The cases and ASRs of incidence, prevalence, mortality and DALYs showed an increased trend over these years, while the MIR of the urological cancers showed a decreased trend. Notably, although the ASRs of DALYs attributed to smoking decreased, the cases to smoking and the cases and ASRs to others showed an increased trend in China from 1990 to 2021. Finally, the burden of these cancers exerts a particularly heavy toll on the older adult population, and the sex differences therein are remarkable. However, these findings should be interpreted with caution due to several limitations. The reliance on GBD 2021 data may lead to potential discrepancies in regional burden estimation, especially for subnational areas. Additionally, the study’s focus on limited risk factors may overlook unmeasured determinants, potentially hindering a comprehensive understanding of cancer etiology. The ecological nature of the data also restricts inferences at the individual level, necessitating future studies based on individual-level datasets to validate these trends and refine risk assessments. Our findings highlight the necessity of implementing effective prevention and intervention measures to target these particular risk factors and safeguard the vulnerable populations in China.

## References

[1] Zi H, Liu MY, Luo LS (2024). Global burden of benign prostatic hyperplasia, urinary tract infections, urolithiasis, bladder cancer, kidney cancer, and prostate cancer from 1990 to 2021. Mil Med Res.

[2] Sung H, Ferlay J, Siegel RL, Laversanne M, Soerjomataram I, Jemal A, Bray F (2021). Global cancer statistics 2020: GLOBOCAN estimates of incidence and mortality worldwide for 36 cancers in 185 countries. CA Cancer J Clin.

[3] Jin YH, Zeng XT, Liu TZ (2022). Treatment and surveillance for non-muscle-invasive bladder cancer: a clinical practice guideline (2021 edition). Mil Med Res.

[4] Ferlay J, Colombet M, Soerjomataram I, Parkin DM, Piñeros M, Znaor A, Bray F (2021). Cancer statistics for the year 2020: an overview. Int J Cancer.

[5] Wang Z, Wang L, Wang S, Xie L (2022). Burden of kidney cancer and attributed risk factors in China from 1990 to 2019. Front Public Health.

[6] Luo LS, Luan HH, Zhang P, Jiang JF, Zeng XT, Huang J, Jin YH (2024). The disease burden of bladder cancer and its attributable risk factors in five Eastern Asian countries, 1990-2019: a population-based comparative study. BMC Public Health.

[7] Zhou H, Hong X, Miao W, Wang W, Wang C, Han R, Zhou J (2024). Exploring prostate cancer incidence trends and age change in cancer registration areas of Jiangsu Province, China, 2009 to 2019. Curr Oncol.

[8] Zeng H, Chen W, Zheng R (2018). Changing cancer survival in China during 2003-15: a pooled analysis of 17 population-based cancer registries. Lancet.

[9] Chow WH, Dong LM, Devesa SS (2010). Epidemiology and risk factors for kidney cancer. Nat Rev Urol.

[10] Jubber I, Ong S, Bukavina L (2023). Epidemiology of bladder cancer in 2023: a systematic review of risk factors. Eur Urol.

[11] Bergengren O, Pekala KR, Matsoukas K (2023). 2022 update on prostate cancer epidemiology and risk factors-a systematic review. Eur Urol.

[12] Pan XF, Wang L, Pan A (2021). Epidemiology and determinants of obesity in China. Lancet Diabetes Endocrinol.

[13] Chan KH, Wright N, Xiao D (2022). Tobacco smoking and risks of more than 470 diseases in China: a prospective cohort study. Lancet Public Health.

[14] Chen X, Giles J, Yao Y (2022). The path to healthy ageing in China: a Peking University-Lancet Commission. Lancet.

[15] (2024). Global incidence, prevalence, years lived with disability (YLDs), disability-adjusted life-years (DALYs), and healthy life expectancy (HALE) for 371 diseases and injuries in 204 countries and territories and 811 subnational locations, 1990-2021: a systematic analysis for the Global Burden of Disease Study 2021. Lancet.

[16] Kocarnik JM, Compton K, Dean FE (2022). Cancer incidence, mortality, years of life lost, years lived with disability, and disability-adjusted life years for 29 cancer groups from 2010 to 2019: a systematic analysis for the Global Burden of Disease Study 2019. JAMA Oncol.

[17] (2024). Global burden and strength of evidence for 88 risk factors in 204 countries and 811 subnational locations, 1990-2021: a systematic analysis for the Global Burden of Disease Study 2021. Lancet.

[18] (2020). Global age-sex-specific fertility, mortality, healthy life expectancy (HALE), and population estimates in 204 countries and territories, 1950-2019: a comprehensive demographic analysis for the Global Burden of Disease Study 2019. Lancet.

[19] Jiang T, Guo H, Liu Y (2024). A comprehensive genetic variant reference for the Chinese population. Sci Bull (Beijing).

[20] Qiao Z, Xiong J, Zhang S, Chen L, Wang J, Chen R (2025). A comparative analysis of global and Chinese trends in the burden of kidney cancer from 1990 to 2021. Sci Rep.

[21] Liu X, Yu C, Bi Y, Zhang ZJ (2019). Trends and age-period-cohort effect on incidence and mortality of prostate cancer from 1990 to 2017 in China. Public Health.

[22] Fitzgerald KN, Motzer RJ, Lee CH (2023). Adjuvant therapy options in renal cell carcinoma - targeting the metastatic cascade. Nat Rev Urol.

[23] Tran L, Xiao JF, Agarwal N, Duex JE, Theodorescu D (2021). Advances in bladder cancer biology and therapy. Nat Rev Cancer.

[24] Bakht MK, Beltran H (2025). Biological determinants of PSMA expression, regulation and heterogeneity in prostate cancer. Nat Rev Urol.

[25] Wang M, Luo X, Xu S (2019). Trends in smoking prevalence and implication for chronic diseases in China: serial national cross-sectional surveys from 2003 to 2013. Lancet Resp Med.

[26] Dai C, Heemers H, Sharifi N (2017). Androgen signaling in prostate cancer. Cold Spring Harb Perspect Med.

[27] Butler EN, Zhou CK, Curry M (2023). Testosterone therapy and cancer risks among men in the SEER-Medicare linked database. Br J Cancer.

[28] Li N, Zhai Z, Zheng Y (2021). Association of 13 occupational carcinogens in patients with cancer, individually and collectively, 1990-2017. JAMA Netw Open.

